# Fully human HER2/cluster of differentiation 3 bispecific antibody triggers potent and specific cytotoxicity of T lymphocytes against breast cancer

**DOI:** 10.3892/mmr.2015.3441

**Published:** 2015-03-05

**Authors:** YAN ZHOU, LAN-TU GOU, ZHI-HUI GUO, HAI-RONG LIU, JIANG-MAN WANG, SHU-XIAN ZHOU, JIN-LIANG YANG, XIAO-AN LI

**Affiliations:** 1 Gastroenterology Tumor and Microenvironment Laboratory, Department of Gastroenterology, The First Affiliated Hospital of Chengdu Medical College, Chengdu, Sichuan 610041, P.R. China; 2 State Key Laboratory of Biotherapy, West China Hospital, Sichuan University, Chengdu, Sichuan 610041, P.R. China

**Keywords:** bispecific antibody, HER2, cluster of differentiation 3, breast cancer, immunotherapy

## Abstract

The use of a bispecific antibody (BsAb) is a promising and highly specific approach to cancer therapy. In the present study, a fully human recombinant single chain variable fragment BsAb against human epidermal growth factor receptor (HER)2 and cluster of differentiation (CD)3 was constructed with the aim of developing an effective treatment for breast cancer. HER2/CD3 BsAb was expressed in Chinese hamster ovary cells and purified via nickel column chromatography. Flow cytometry revealed that the HER2/CD3 BsAb was able to specifically bind to HER2 and CD3-positive cells. HER2/CD3 BsAb was able to stimulate T-cell activation and induce the lysis of cultured SKBR-3 and BT474 cells in the presence of unstimulated T lymphocytes. HER2/CD3 BsAb efficiently inhibited the growth of breast cancer tissue by activating and inducing the proliferation of tumor tissue infiltrating lymphocytes. Therefore, HER2/CD3 BsAb is a potent tool which may be a suitable candidate for the treatment of breast cancer.

## Introduction

Breast cancer is a serious threat to the health of females worldwide. Approximately 120 million individuals develop breast cancer each year, resulting in 500,000 fatalities (1,2). Treatment regimens for breast cancer include chemotherapy, endocrine therapy and molecular targeted therapy. Breast cancer chemotherapy mainly uses drugs developed in the 1970s, including cyclophosphamide, methotrexate, fluorouracil and taxane paclitaxel (3,4). Endocrine treatment of breast cancer is performed via the use of estrogen antagonists and aromatase inhibitors (5). Breast cancer treatment through molecular targeting agents constitute drugs that target the human epidermal growth factor receptor (HER) family, including trastuzumab/herceptin, lapatinib and others. Angiogenesis inhibitors (bevacizumab/avastin) have also been used in clinical trials (6,7).

The HER family includes HER1 (epidermal growth factor receptor; EGFR), HER2, HER3 and HER4, which are transmembrane tyrosine kinase receptors. They are mainly involved in cell growth, proliferation and signal transduction (8). HER-2/neu, also known as c-erb-B2, is a proto-oncogene, which is expressed in a variety of tumors and metastases, including breast cancer, head and neck cancer, colorectal cancer and ovarian cancer (9–12). A study has confirmed that 20–30% of patients with breast cancer have a high expression of HER-2 and that overexpression of HER-2 in patients correlates with a poor prognosis and resistance to cytotoxic chemotherapeutic drugs (13). The anti-HER2 monoclonal antibody herceptin was the first to be used in breast cancer treatment and has been widely applied in the treatment of breast cancer in China (14). At present, herceptin is used as a first-line treatment against breast cancer. Herceptin is a humanized immunoglobulin (Ig) G1 monoclonal antibody, which inhibits the signal transduction pathways of cell growth and mediates the antibody-dependent cellular cytotoxicity mechanisms to inhibit tumor growth (15). In clinical trials, the remission rate of adriamycin and cyclophosphamide (AC) chemotherapy is 42% for metastatic breast cancer and the combinatorial therapy of herceptin with the AC program is associated with an increased remission rate of 65% (16). Additionally, in therapies combining herceptin with paclitaxel, the complete remission rate is 57%, which is higher than that with therapies using only paclitaxel (25%) (16). However, herceptin is a chimerized murine/human anti-HER2 IgG1 antibody and therapies involving herceptin do have disadvantages, including drug resistance, frequent side effects and requirements of high concentrations of the antibody (17).

An efficient, genetically engineered bispecific antibody (BsAb) was developed in previous years, which utilizes a combination of antibodies targeting the surface of T cells (cluster of differentiation; CD3) and tumor surface antigens (epithelial cell adhesion molecule, CD19 and HER2) (18). CD3 is the main component of the T-cell receptor/CD3 complex on the T-cell surface and is the key molecule required for the activation of T lymphocytes. The CD3 antibody binds to CD3 and induces the activation of T lymphocytes, which is followed by the release of perforin, granzyme factor and cytokines, which are important in eliminating tumor cells (19). Therefore, the cytotoxic T cell is the most important effector of cellular immunity and T cells are critical for the clearance of tumor cells *in vivo*. The BsAb activates the cytotoxicity of T cells by specifically binding to the T cells and tumor cells, thereby eliminating the tumor cells. In addition, the required clinical dose of the BsAb is extremely low at only 1/500 of that required for herceptin (20). Previous studies have confirmed that BsAbs targeting CD3 and the tumor antigens may activate cytotoxic T cells to specifically target and eliminate tumor cells (21).

In the present study, a fully human recombinant single-chain HER2/CD3 BsAb was constructed. To the best of our knowledge, the present study was the first to describe a fully human HER2/CD3 BsAb with high levels of anti-tumoral activity. The recombinant BsAb molecule was expressed and secreted in a fully active form by mammalian cells. The binding characteristics of the HER2/CD3 BsAb and its ability to stimulate T-cell activation and to induce lysis of BT474 and SKBR-3 cells were assessed. In addition, the ability of the HER2/CD3 BsAb to inhibit the growth of breast cancer tissue and to induce the proliferation of tumor tissue-infiltrating lymphocytes was examined. The results of the present study indicated that HER2/CD3 BsAb may be a suitable candidate for the treatment of breast cancer.

## Materials and methods

### Cell lines and breast cancer tissue

The HER2-positive cell lines BT474 and SKBR-3, the CD3-positive T-cell line Jurkat and the HER2-negative cell line MDA-MB-231 were all obtained from the American Type Culture Collection (ATCC; Manassas, VA, USA). Chinese hamster ovary cells (CHO) used for expressing BsAbs were also obtained from the ATCC. Human breast cancer tissues were obtained from breast cancer patients undergoing biopsy at the First Affiliated Hospital of Chengdu Medical College (Chengdu, China) in march 2013. Inclusion criteria were that the tumors were HER2 positive, first operation patients without medication and radiation. The protocol of the present study was approved by the Institutional Ethics Committee of Chengdu Medical College. Informed consent for the present study was received from all patients prior to the commencement of the experiments.

### Construction of single-chain BsAb fragments

The single-chain variable fragment (scFv) of HER2 and CD3 antibodies were cloned from the vectors pET-26a-HER2 and pET-26a-CD3, respectively. These two vectors have been previously established in our laboratory (Gastroenterology Tumor and Microenvironment Laboratory, Chengdu, China) and the gene sequences of the HER2 and CD3 antibodies were screened from the human natural antibody library (22). The anti-HER2 scFv fragment and anti-CD3 scFv fragment were linked with (G_4_S)_3_ by overlapping polymerase chain reaction to produce the recombinant protein VH(HER2)-VL(HER 2)-(G_4_S)_3_-VL(CD3)-VH(CD3), with an inserted interleukin (IL)-2 signal peptide at the N-terminus and a histidine tag at the C-terminus. Subsequently, the entire BsAb molecule was cloned into the expression vector pcDNA3.1 (Stratagene, La Jolla, CA, USA).

### BsAb expression and purification

CHO cells (2×10^5^/1 ml) were cultured in six-well flat-bottom plates and transfected with 2 *μ*g plasmid DNA and 4 *μ*g Lipofectamine 2000 (Invitrogen Life Technologies, Carlsbad, CA, USA). The transfection medium was removed after 4 h and cells were incubated at 37°C with fresh medium. CHO cells, which stably expressed HER2/CD3 BsAb were screened using G418 antibiotics (Gibco Life Technologies, Grand Island, NY, USA) for 48 h. The culture supernatant was collected and purified by immobilized nickel metal affinity chromatography (ÄKTA explorer, GE Healthcare, Little Chalfont, UK) on Ni-charged chelating sepharose (Amersham Pharmacia Biotech, GE Healthcare).

### Flow cytometry

BT474, SKBR-3 and Jurkat cells as well as peripheral blood mononuclear cells (PBMCs; provided by State Key Laboratory or Biotherapy) were used for detection of antibody binding, while the HER2/CD3 negative cell line MDA-MB-231 was used as a negative control. A total of 1×10^6^ cells were washed with phosphate-buffered saline (PBS; 137 mmol/l NaCl, 2.7 mmol/l KCl, 10 mmol/l Na_2_HPO_4_ and 2 mmol/l KH_2_PO_4_) and incubated in 100 *μ*l HER2/CD3 BsAb (100 *μ*g/ml in PBS) for 30 min at room temperature and then washed twice with PBS. Fluorescein isothiocyanate-conjugated antibody against the His-tag (Abcam, Cambridge, UK) was used for detecting the BsAb. The His-tag antibody (ab1206) is a rabbit polyclonal IgG, which only reacts with human proteins. The antibody was diluted at 1:200 and added to the cells for 30 min at room temperature. Cells were analyzed using fluorescence-activated cell sorting (CytoFLEX; Beckman-Coulter, Pasadena, CA, USA).

### Induction of T-cell activation

Freshly prepared PBMCs (2×10^6^ cells/ml) were added to each well of a six-well flat-bottom plate (Molecular Devices, Sunnyvale, CA, USA). Each well contained 2 ml RPMI 1640 (HyClone, Logan, Utah, USA) with 10% fetal calf serum (FCS) only (control wells), or with 10% FCS and orthoclone OKT3 (30 ng/ml; Wuhan Institute of Biological Products, Wuhan, China) or with 10% FCS and HER2/CD3 BsAb (10 ng/ml). PBMCs were incubated for 24 h and the activation of PBMCs was measured using flow cytometric analysis. The expression levels of CD25 and CD69 on T cells were detected by flow cytometry to evaluate the T-lymphocyte activation ability of HER2/CD3 BsAbs.

### Luminex liquid chip analysis

A luminex liquid chip array was used to determine the release of inflammatory cytokines IL-2, IL-4, tumor necrosis factor (TNF)-α and interferon (IFN)-γ from PBMCs induced by HER 2/CD3 BsAb. A human MultiAnalyte Profiling Base kit (R&D, Minneapolis, Minnesota, USA) was used for detection. Freshly prepared PBMCs (2×10^6^ cells/ml) were added to each well of a 96-well flat-bottom plate. Each well contained 100 *μ*l complete media alone (control wells), or with complete media containing 1 *μ*g/ml CD28 Ab (TGN1412; eBioscience, San Diego, CA, USA), CD3 Ab OKT3 or HER2/CD3 BsAb. Each assay was performed in triplicate. The PBMCs were incubated at 37°C under 5% CO_2_ for 72 h and 50-*μ*l aliquots of media were collected for the liquid chip array. Briefly, the diluted microparticle mixture was resuspended and 50 *μ*l of the mixture was added to each well of the microplate. Subsequently, 50 *μ*l of the standard or sample was added to each well and incubated for 3 h at room temperature using a vacuum manifold device designed to accommodate a microplate. Subsequently, 50 *μ*l diluted biotin antibody cocktail was added to each well and the plate was incubated for 1 h at room temperature, whilst agitated at 45 × g. Diluted streptavidin-phycoerythrin (50 *μ*l) was added to each well and incubated for 30 min at room temperature, whilst agitated at 500 rpm. The microparticles were resuspended by adding 100 *μ*l wash buffer to each well and incubated for 2 min, whilst agitated at 500 rpm. The fluorescence signal was read using a Luminex-100 analyzer (Luminex Corp., Austin, TX, USA) within 90 min.

### Cytotoxicity assay

The HER2-positive cell lines BT474 and SKBR-3 were used as target cells and the MDA-MB-231 cells were used as negative controls. Cytotoxicity was measured using a CytoTox 96^®^ Non-Radioactive Cytotoxicity assay kit (Promega, Madison, Wisconsin, USA) using RPMI 1640 complete medium with 5% FCS in a round-bottom 96-well plates. Briefly, PBMCs were added as effector cells to each well at gradient concentrations, followed by the addition of the target cells (1×10^4^). HER2/CD3 BsAb (100 ng/ml) was then added to achieve final effector cell to target cell (E:T) ratios of 100:1, 50:1, 10:1 and 1:1. The cell mixtures were incubated at 37°C under 5% CO_2_ for 4 h, following which 50 *μ*l aliquots of media were transferred to fresh 96-well flat-bottom plates for the LDH-release assay. The percentage of cell lysis was calculated as the specific release (%) = (experimental release − effector spontaneous release − target spontaneous release) / (target maximum release − target spontaneous release) × 100. Each assay was performed in triplicate.

### Primary culture of HER2-positive breast cancer tissue with HER2/CD3 BsAb

Primary cultures of breast cancer tissue samples for detecting the activity of HER2/CD3 BsAb were initiated by collecting tissue samples of HER2-positive breast cancer from six patients under sterile conditions. The tumor tissues were washed with saline and the fatty tissues and necrotic tissues surrounding the tumor tissue were removed, following which the samples were cut into pieces of 4–8 mm^3^. The tissue samples were weighed and divided into three groups of equal weight randomly. One group was inoculated with RPMI 1640 medium alone and the other group was inoculated with RPMI 1640 medium containing 0.1 *μ*g/ml HER2/CD3 BsAb or 1 *μ*g/ml HER2/CD3. The tissue samples were incubated at 37°C under 5% CO_2_ for five days. On the third day of the incubation period, one tissue sample each was removed from the control group and the experimental groups for hematoxylin and eosin (HE; Beyotime Institue of Biotechnology, Inc., Shanghai, China) staining to determine the proliferation of tumor-infiltrating T cells. On the fifth day, images were captured (Nikon D90 camera; Nikon, Tokyo, Japan) of the remaining tissue samples and their weights were measured. The changes in the volume and weight of the tissue samples were used as measures of therapeutic efficacy.

### Statistical analysis

Values are expressed as the mean ± standard deviation of at least three independent experiments. Differences between the treatment groups in the cytotoxicity assays and tumor tissue weight were analyzed using analysis of variance. P<0.05 was considered to indicate a statistically significant difference. All statistical analyses were calculated using SPSS 16.0 (SPSS, Inc., Chicago, IL, USA) software.

## Results

### Preparation and binding properties of HER2/CD3 BsAb

**Figure 1:**
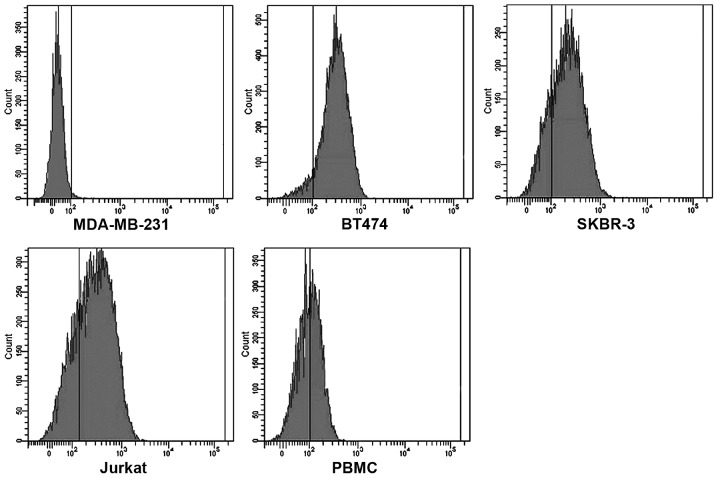
Binding specificity of HER2/CD3 BsAb. Flow cytometric analysis of HER2/CD3 BsAb binding to HER2-positive BT474 and SKBR-3 cells, CD3-positive Jurkat and PBMC cells and HER2-/CD3-negative MDA-MB-231 cells. BsAb, bispecific antibody; CD, cluster of differentiation; HER, human epidermal growth factor receptor; PBMC, peripheral blood mononuclear cell.

In the recombinant plasmid pcDNA3.1, the scFv fragments were linked with the (G _4_S)_3_ linker in the format VH (HER2)-VL(HER2)-(G_4_S)_3_-VL(CD3)-VH(CD3)-6xHis. The IL-2 signal peptide upstream of the HER2/CD3 BsAb directed the HER2/CD3 BsAb to be secreted into the supernatant. The CHO cell culture supernatant was passed through an immobilized nickel metal affinity chromatography column and the HER2/CD3 BsAb eluted from the Ni-NTA column at 300 mM imidazole as a distinct peak. The purified HER2/CD3 BsAb was subjected to 10% SDS-PAGE and analyzed using western blotting. The protein migrated with an apparent molecular mass of 57 kDa, consistent with the theoretical molecular weight. In addition, HER2/CD3 BsAb bound specifically to the HER2-positive BT474 and SKBR-3 cells, as well as CD3-positive Jurkat and PBMCs cells; however, there was no detectable binding to MDA-MB-231 cells that express neither HER2 nor CD3 (Fig. 1). These results indicated that the HER2/CD3 BsAb specifically bound to HER2 and CD3.

### HER2/CD3 BsAb induces T-cell activation

**Figure 2:**
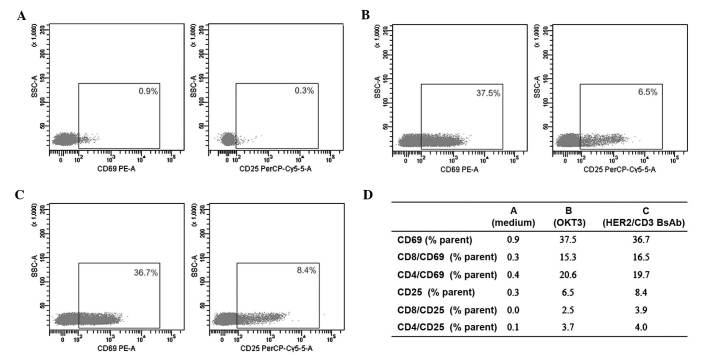
CD69 and CD25 upregulation induced by the HER2/CD3 BsAb in CD4/CD8 T cells. The PBMCs were treated with medium alone, OKT3 (30 ng/ml) or CD19/CD3 BsAb (10 ng/ml) for 24 h and the expression levels of CD69 and CD25 were measured. (A) Expression levels of CD69 and CD25 on T cells when PBMCs were treated with medium alone. (B) CD69 and CD25 on the T-cell surface following stimulation with CD3 antibody OKT3. (C) Upregulation of CD69 and CD25 on T cells induced by HER2/CD3 BsAb. (D) Summary of expression levels of CD69 and CD25 on CD4 or CD8 T cells induced by medium alone, OKT3 or HER2/CD3 BsAb. BsAb, bispecific antibody; CD, cluster of differentiation; HER, human epidermal growth factor receptor; PBMC, peripheral blood mononuclear cell.

The expression of CD25 and CD69 on T cells is rapidly upregulated upon activation. To evaluate the ability of HER2/CD3 BsAb to activate T lymphocytes, the expression of CD25 and CD69 on T cells was monitored using flow cytometry. The results demonstrated that the rate of CD25-expressing cells was 8.4% and the rate of CD69-expressing cells was 36.7% among cells treated with HER2/CD3 BsAb, compared with those in the PBS-treated group. In CD4 and CD8 T cells from the HER2/CD3 BsAb-treated group, the rate of CD25-expressing cells was 4.0 and 3.9%, respectively, while the rate of CD69-expressing cells was 19.7 and 16.5%, respectively. In cells treated with OKT3, the rate of CD25-expressing cells was 6.5% and the rate of CD69-expressing cells was 37.5%. In CD4 or CD8 T cells from the OKT3-treated group, the expression proportion of CD25 was 3.7 and 2.5%, respectively, and that of CD69 was 20.6 and 15.3%, respectively (Fig. 2). These data indicated that T-cell activation by HER2/CD3 BsAb was similar to that by OKT3, without any pre-stimulus to induce T-cell activation.

### HER2/CD3 BsAb induces release of cytokines from PBMCs

**Figure 3:**
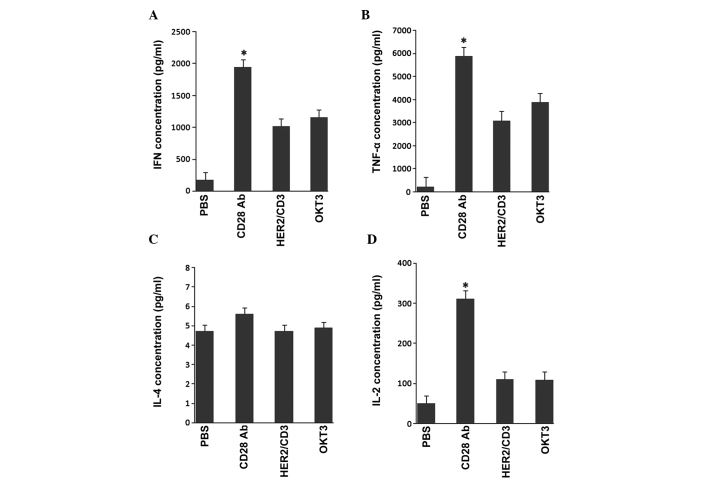
Liquid chip analysis of cytokines released by activated T lymphocytes. PBMCs were treated with PBS, 1 *μ*g/ml CD28 Ab, CD3 Ab OKT3 or HER2/CD3 BsAb for 72 h. Release of the cytokines (A) IFN-γ, (B) TNF-α, (C) IL-4 and (D) IL-2 was measured. ^*^P<0.01, vs. other three groups. Ab, antibody; BsAb, bispecific antibody; CD, cluster of differentiation; HER, human epidermal growth factor receptor; PBMC, peripheral blood mononuclear cell; IFN, interferon; TNF, tumor necrosis factor; IL, interleukin; PBS, phosphate-buffered saline.

Secretion of cytokines, including TNF-α, IFN-γ, IL-4 and IL-2 from PBMCs induced by HER2/CD3 BsAb, CD3-Ab OKT3 and CD28-Ab were determined under similar conditions to those described above (2×10^6^ cells/ml PBMCs, 1 *μ*g/ml CD28 Ab, CD3 Ab OKT3 or HER2/CD3 BsAb incubated for 72 h). The results revealed that the release of TNF-α, IFN-γ and IL-2 induced by CD28-Ab was significantly higher than that induced by OKT3 and HER2/CD3 BsAb, whereas the secretion of cytokines induced by OKT3 and HER2/CD3 BsAb were comparable. No significant differences were identified between OKT3, CD28-Ab and HER2/CD3 BsAb in their ability to induce the release of IL-4 (Fig. 3).

### HER2/CD3 BsAb mediates specific cytotoxicity against breast cancer cells

**Figure 4:**
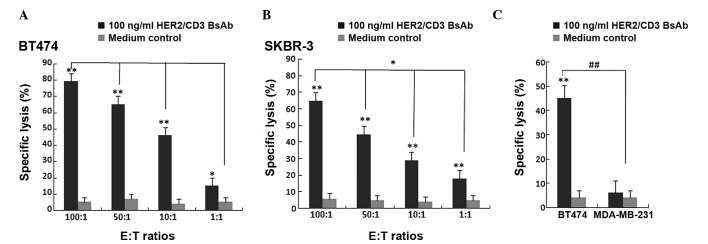
Cytotoxic effect of HER2/CD3 BsAb. Cytotoxicity of HER2/CD3 BsAb as measured using a lactate dehydrogenase-release assay. Cells were incubated for 4 h with 100 ng/ml HER2/CD3 BsAb. Primary human PBMCs and (A) BT474 or (B) SKBR-3 cells were incubated at various E:T ratios (100:1, 50:1, 10:1 and 1:1). Values are expressed as the mean ± standard deviation. ^*^P<0.01, ^**^P<0.001, compared with medium control. (C) Primary human PBMCs and BT474 cells or MDA-MB-231 cells were incubated at an E:T ratio of 10:1. Values are expressed as the mean ± standard deviation. ^##^P<0.001, ^**^P<0.001, compared with medium control. BsAb, bispecific antibody; CD, cluster of differentiation; HER, human epidermal growth factor receptor; E:T, effector cell to target cell; PBMC, peripheral blood mononuclear cell.

The cytotoxic activity of HER2/CD3 BsAb against the breast cancer cell lines BT474 and SKBR-3 were measured using the LDH-release assay. Unstimulated PBMCs were added as effector cells to target BT474 and SKBR-3 cells at E:T ratios of 100:1, 50:1, 10:1 and 1:1. In parallel wells, HER2-negative breast cancer MDA-MB-231 cells were used as a negative control. HER2/CD3 BsAb may significantly induce BT474 and SKBR-3 cell death at all E:T ratios without pre-stimulation, while no cytotoxic activity was observed when MDA-MB-231s were used as target cells. The cytotoxic activity of HER2/CD3 BsAb was significantly increased at higher E:T ratios (Fig. 4). These results suggested that HER2/CD3 BsAb-mediated cytotoxic effects were HER2-specific and dependent on T cells without pre-stimulation.

### Inhibition of breast cancer cell growth by HER2/CD3 BsAb

**Figure 5:**
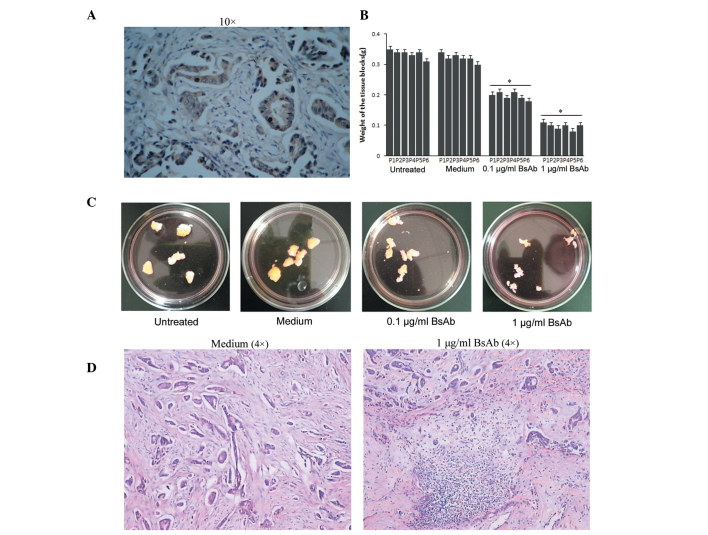
HER2/CD3 BsAb treatment inhibits breast cancer cell growth. (A) Detection of HER2 in breast tumor tissues using immunohistochemical staining. (B) Weight of breast cancer tissue samples of six patients, treated as above. P1, patient 1 and so on. P<0.01, vs. other three groups. (C) Images displaying the volume of HER2-positive breast cancer tissue samples treated with RPMI-1640 medium alone, 0.1 *μ*g/ml HER2/CD3 BsAb or 1 *μ*g/ml HER2/CD3 BsAb. (D) Hematoxylin and eosin staining of breast cancer tissue samples treated with RPMI-1640 medium alone or 1 *μ*g/ml HER2/CD3 BsAb. BsAb, bispecific antibody; CD, cluster of differentiation; HER, human epidermal growth factor receptor.

In order to detect the anti-tumoral activity of HER2/CD3 BsAb, the HER2-positive breast cancer tissue samples from six patients were treated with RPMI 1640 medium alone or RPMI 1640 medium containing 0.1 *μ*g/ml or 1 *μ*g/ml HER2/CD3 BsAb and the tumor growth was determined using volume and weight measurements. The colon cancer tissues selected for incubation with HER2/CD3 BsAb were HER2-positive (Fig. 5A). The results demonstrated that treatment with 0.1 *μ*g/ml or 1 *μ*g/ml HER2/CD3 BsAb significantly inhibited breast tumor cell growth compared with that of vehicle-treated cancer tissue samples. A significant reduction in the growth of HER2-positive breast cancer cells from six patients was observed. The volume of the tissue samples treated with HER2/CD3 BsAb was significantly lower than that of the vehicle-treated tissue. With the increase in the concentration of HER2/CD3 BsAb, the weight of the tissue samples decreased. The results revealed that the HER2/CD3 BsAb significantly inhibited the growth of HER2-positive breast tumor cells (Fig. 5B and C). HE staining demonstrated that HER2/CD3 BsAb was able to induce the proliferation of tumor tissue-infiltrating lymphocytes (Fig. 5D). This indicated that the HER2/CD3 BsAb-mediated anti-tumoral effects were HER2-specific and dependent on the tumor tissue-infiltrating lymphocytes.

## Discussion

The use of BsAbs (mouse origin) for the treatment of breast cancer has been observed to be effective *in vitro* and *in vivo* (23,24). In the present study, a fully human recombinant single chain BsAb, which targeted CD3 and HER2, was constructed. Recombinant HER2/CD3 BsAb acted as a powerful stimulator of T-cell activation and induced cytotoxicity in breast tumor BT474 and SKBR-3 cells in the presence of T cells. HER2/CD3 BsAb may also efficiently inhibit the growth of HER2-positive breast tumor samples by activating and inducing the proliferation of tumor tissue-infiltrating lymphocytes. The anti-tumoral effects of HER2/CD3 BsAb required no pre-stimulation with human PBMCs, even at low doses of HER2/CD3 BsAb (0.1 *μ*g/ml). Furthermore, the cytokine release assay revealed that HER2/CD3 BsAb was not similar to the anti-CD28 agonist antibody (TGN1412). These results indicated that the HER2/CD3 BsAb is a potent candidate treatment for patients with HER2 positive breast cancer.

The pharmacodynamic evaluation of BsAbs *in vivo* is a complex process. Conventionally, the evaluation is mainly performed through the establishment of tumor animal models followed by treatment with BsAbs and lymphocytes. In addition, the changes in tumor weight and survival time may be used as measures of therapeutic efficacy (25,26). However, this method does have certain limitations. Firstly, the type of animal model and treatment method may markedly affect the treatment efficacy of BsAbs and therefore, it is difficult to isolate the effects of the clinical condition of the tumor from the animal model and treatment method. Secondly, a large volume of fresh blood is necessary for extracting the lymphocytes required for the experiment. In the present study, fresh breast cancer tissue culture was used to evaluate the anti-tumoral activity of BsAbs. Samples of breast cancer tissue which had been surgically removed were collected and inoculated with HER2/CD3 BsAb. Changes in the volume and weight of the tissue samples were used as measures of therapeutic efficacy. It was observed that with an increase in the concentration of HER2/CD3 BsAb, the weight of the tissue samples decreased. The advantage of this method is the relatively simple procedure, reproducibility, controllability and a more accurate reflection of the *in vivo* physiological condition in patients.

The anti-CD28 agonist antibody (TGN1412) has received attention due to its marked adverse reactions in Phase I clinical trials (27). TGN1412 is able to induce T-cell activation to further activate the immune system by combining with CD28 on the cell surface of T cells. In the first human clinical trial, within 12–16 h following injection with TGN1412, all subjects developed symptoms of pulmonary infiltration, acute lung injury, diffuse intravascular coagulation and renal failure. In the first six to eight days after TGN1412 injection, two subjects exhibited intense cardiovascular injury, acute respiratory distress syndrome and multiple organ failure. Serum analyses of volunteers injected with TGN1412 revealed a significant increase in the levels of inflammatory cytokines, including TNF-α and IFN-γ as well as IL-1β, −2, −4, −6, −8 and −10 levels. Cytokines direct the function and activity of the immune system. When the expression levels of cytokines show sudden and marked changes, a series of emergency commands are sent to the lymphocytes, which leads to an immediate induction of T-cell activation. Activated lymphocytes migrate to the various tissues and organs, triggering an acute inflammatory reaction, attacking the system and organs, finally causing multiple organ failure, which was observed within the subjects in the TGN1412 trial. Simultaneously, as the bone marrow and the hematopoietic system are not able to produce a sufficient number of lymphocytes in a short period of time, peripheral blood lymphocyte depletion occurs.

HER2/CD3 BsAb belongs to the same category of immune agonist antibodies as TGN1412 and identifies and activates the immune cells to eliminate tumor cells. Due to the adverse reaction of TGN1412, it is important to detect inflammatory cytokines. In the present study, the quantity of TNF-α, IFN-γ, IL-4 and IL-2 induced by HER2/CD3 BsAb, monoclonal antibody to CD3-OKT3 and monoclonal antibody to CD28 were determined under the same conditions. The results demonstrated that the release of TNF-α, IFN-γ and IL-2 induced by the CD28 monoclonal antibody were significantly higher than that induced by OKT3 and HER2/CD3 BsAb, while the release of TNF-α, IFN-γ and IL-2 induced by OKT3 was similar to that induced by HER2/CD3 BsAb. No significant difference was identified between OKT3, CD28 monoclonal antibody and HER2/CD3 BsAb in stimulating the release of IL-4. Considering that OKT3 is listed as a drug that is safe and reliable in clinical treatment and that the CD19/CD3 BsAb antibody has exhibited a potent anti-tumoral effect and qualified as safe in Phase I clinical trials (20), HER2/CD3 BsAb is also expected to be safe in clinical treatment.

Currently, the antibody drugs available for cancer treatment are either chimeric antibodies or humanized antibodies, including rituxan and herceptin (28,29). The main limitation of these antibodies is the marked immunogenicity that induces a human anti-mouse antibody (HAMA) response. The HAMA response may cause allergic reactions and the neutralization of the exogenously administered antibodies, reducing their efficacy. In the present study, a fully human BsAb was constructed, which may effectively decrease the immunogenicity of the BsAb and thus enhance its efficacy and reduce the side effects.

In conclusion, the fully human recombinant scFv BsAb against HER2 and CD3 was constructed in the present study, which was shown to be a highly potent inducer of T-cell activation. HER2/CD3 BsAb may induce the lysis of cultured SKBR-3 and BT474 cells in the presence of unstimulated T lymphocytes. In addition, the HER2/CD3 BsAb efficiently inhibited the growth of breast cancer tissue samples by activating and inducing the proliferation of tumor tissue infiltrating lymphocytes. Furthermore, when incubated with PBMCs, HER2/CD3 BsAb did not act similarly to the anti-CD28 agonist antibody (TGN1412), which previously led to a life-threatening cytokine storm in the first human trials. This HER2/CD3 BsAb is fully human and only a low dose is required for significant therapeutic efficacy. It is thus a potential candidate for the clinical treatment of patients with HER2-positive breast cancer.
